# Gitelman’s Syndrome in Pregnancy With Adverse Foetal Outcome: A Case Report

**DOI:** 10.7759/cureus.34791

**Published:** 2023-02-09

**Authors:** Rehana Najam, Yugantika Tomar, Mrinalini Singh

**Affiliations:** 1 Obstetrics and Gynaecology, Teerthanker Mahaveer Medical College and Research Center, Moradabad, IND

**Keywords:** foetal outcome, metabolic alkalosis, hypocalciuria, hypomagnesemia, hypokalemia, gitleman's syndrome

## Abstract

Gitelman’s syndrome (GS) is a disorder characterized by hypokalaemia, hypomagnesemia, hypocalciuric and metabolic alkalosis. Despite the fact that it affects women of child-bearing age, only limited information is available regarding its impact on maternal and foetal outcome. We present the case of an un-booked and un-investigated 23-year-old primigravida who presented with chief complaints of vomiting and loose stools. The patient also complained of absent foetal movements in the last 12 hours. Investigations revealed hypokalaemia and hypomagnesemia and ultrasound revealed intra-uterine foetal demise. The patient was symptomatically relieved after electrolyte correction. Scarce reports on Gitelman’s syndrome in pregnancy have been documented with the majority of cases showing positive outcomes for the foetus. We hereby present a report of a primigravida with Gitelman’s syndrome and foetal loss which is considered uncommon.

## Introduction

Maintaining an electrolyte as well as fluid balance is a necessity for a successful pregnancy. Disturbance in fluid and electrolyte balance has been related to an upsurge in the chances of morbidity and mortality, particularly among the elderly [1]. Dehydration can be caused by a variety of factors, including Gitelman’s syndrome, which is discussed in detail here.

Gitelman’s syndrome (familial hypokalaemia-hypomagnesemia) is characterized by hypokalaemic metabolic alkalosis, hypomagnesemia, and inadequate urinary calcium excretion. It is a relatively prevalent but overlooked cause of hypokalaemia. It is a hereditary renal tubular illness with an incidence of 1-10/40,000 [2], that was initially discovered in 1966 [3,4]. It might be because of the inactivation of mutations in the *SLC12A3* gene, which codes for the apical sodium chloride cotransporter and is found on chromosome 16 (16q13), leading to salt wasting, hypokalaemia, alkalosis, and hypocalciuria [5]. As a result of increased sodium delivery to the cortical collecting duct, sodium reabsorption by the epithelial sodium channel increases, which is counterbalanced by potassium loss, resulting in hypokalaemia.

The increased distal exchange of magnesium ions for sodium ions causes hypomagnesaemia. Salt cravings, exhaustion, and postural hypotension are all common symptoms of this condition. Gitelman’s syndrome has been related to a higher chance of miscarriage as well as Intra Uterine Growth Restriction (IUGR) during pregnancy along with significant maternal morbidity due to electrolyte imbalance concerns [6-9].

## Case presentation

A 23-year-old primigravida at 37 weeks period of gestation, un-booked and un-investigated, presented to the emergency with chief complaints of absent fetal movements in the last 12 hours, vomiting for five days (5-6 episodes per day), loose stools for three days, itching over palms and soles for two weeks. Her vitals were normal with a mild degree of fever. There was no jaundice. Abdominal findings revealed no focal tenderness or guarding, the uterus was relaxed and corresponding to 36 weeks period of gestation, cephalic presentation, and on auscultation, the foetal heart sound could not be localized clinically. Per vaginal examination was done and the patient was found to be in the latent phase of labour. 

Laboratory examination including a complete haemogram, kidney and liver function test, and urine analysis was performed. Arterial blood gas revealed pH 7.5, pCO2 43 mm of Hg, Na+ 135 mEq/L, K 1.92 mEq/L, chloride 99 mEq/L, bicarbonate 31.7 mmol/L. The electrocardiogram revealed PR interval prolongation of 0.24 seconds, flat t waves, and prominent U waves. Her serum potassium level was 2.5 mEq/L, and her serum Mg level was 1.2 mEq/L, according to her first chemistry panel (Table 1).

**Table 1:** Laboratory findings on admission SGOT: Serum glutamic-oxaloacetic transaminase; SGPT: Serum glutamic-pyruvic transaminase

PARAMETERS	RESULTS	NORMAL RANGE
Sodium (mEq/L)	134	135-155
Potassium (mEq/L)	2.5	3.5-5.5
Chloride (mEq/L)	99	90-120
Calcium (mg/dl)	9.3	8.2-10.5
Magnesium (mEq/L)	1.2	1.4-4
Phosphorus (mg/dl)	4.7	2.5-6.2
Urea (mg/dl)	52	13-45
Creatinine (mg/dl)	3.13	0.5-1.2
Uric Acid (mg/dl)	8.6	3.5-7.2
Albumin (g/dl)	4.3	3.5- 5
HbA1c (%)	4.8	< 5.7%
SGOT (U/L)	20	5-40
SGPT (U/L)	10	5-40
ARTERIAL BLOOD GAS ANALYSIS		
pH	7.5	
pCO2	43 mm of Hg	
Na^+^	135 mEq/L	
K^+^	1.92 mEq/L	
Bicarbonate	31.7 mmol/L	
Cl^-^	99 mEq/L	

Her ultrasound revealed a single intrauterine foetus of 34 weeks 6 days with absent cardiac activity suggestive of intra-uterine foetal demise. Injectable ondansetron was used to reduce nausea and vomiting, and racecadotril was used to manage loose stools, but electrolyte imbalances were critically low despite the recovery of hyperemesis. The first line of treatment was intravenous (IV) isotonic saline and parenteral potassium. Labour induction of intra-uterine fetal demise was done using two doses of cerviprime gel six hours apart, followed by two doses of tablet misoprostol 50 mcg four hours apart. The patient was then taken up for emergency lower segment cesarean section (LSCS) in view of obstructed labour. Serum potassium level was checked continuously during her hospitalisation (Table 2). 

**Table 2:** Serial potassium values

DAY OF HOSPITALISATION	SERUM POTASSIUM (mEq/L)
DAY 0	2.5
DAY 1	2.7
DAY 2	2.7
DAY 3	2.9
DAY 4	2.6
DAY 5	3.2
DAY 6	3.6

This patient was readmitted many times during her first trimester of pregnancy for recurrent nausea and vomiting, each time with severe hypokalemia and hypomagnesemia, and her care was continued according to the previous plan. She was relatively asymptomatic except for nausea and emesis, and she denied muscle cramps, weakness, palpitations, or syncopal attacks.

## Discussion

Renal potassium wasting is promoted by physiological changes during pregnancy, while blood potassium level is controlled in the physiologic array due to an increase in progesterone level that opposes kaliuresis. This compensatory mechanism is rapidly overcome in the presence of Gitleman syndrome, resulting in severe hypokalemia.

Maintenance of fluid balance in pregnant women with Gitleman syndrome might be a challenging task. Despite various physiological changes, such as volume expansion, increased renal blood flow, increased glomerular filtration rate (GFR), and activation of the renin-angiotensin-aldosterone axis, that contribute to renal potassium squandering during pregnancy, potassium homeostasis typically remains normal. Increased progesterone levels, which fight kaliuresis, maintain normal serum potassium levels [5].

As was the case in our instance, moderate diarrhoea, vomiting, and pregnancy are common precipitating events. An ideal environment for the development of severe symptomatic hypokalaemia is created in pregnancy by the surge in demand for magnesium and potassium accompanied by increased urine loss. The typical finding of hypomagnesaemia along with metabolic alkalosis, hypokalaemia, hypocalciuria, and hypermagnesuria in 24-hour urine samples serves as the basis for the diagnosis. Genetic testing and the identification of the *SCL12A3* gene mutation are the sole methods for diagnosing this condition [10].

**Table 3:** Summary of cases of Gitelman syndrome during pregnancy

Author	Symptoms	Serum Potassium values	Mode of Delivery	Pregnancy Outcome	Treatment	Gestational Complication
Basu et al. [6]	Easy fatigability in 3^rd^ trimester	2.5 mmol/L	Induced vaginal delivery	Healthy female child of 2.4 kg	Potassium and Magnesium supplementation	Oligohydramnios
Jones et al. [7]	New onset seizures	∼3.2 mEq/L	Induced vaginal delivery	Healthy male child of 2.5 kg	Potassium and Magnesium supplementation	Oligohydramnios
Talaulikar et al. [8]	Fatigue, Muscle cramps	2.7 mmol/L	Spontaneously delivered vaginally	Healthy male child of 2.6 kg	Potassium and Magnesium supplementation	None
De Haan et al. [11]	Fatigue, Muscle weakness	Not available	Delivery by caesarean section	Healthy female child of 2.9 kg	Intravenous Potassium and Magnesium supplementation	Oligohydramnios, Intra uterine growth restriction
De Arriba et al. [12]	Limb paraesthesia, myalgia	2.4–2.8 mEq/L	Elective caesarean section.	Healthy female child of 3 kg	Spironolactone, Potassium and Magnesium supplementation	Oligohydramnios
Daskalakis et al. [13]	Fatigability, muscle weakness and tenderness in bilateral lower limbs	Not available	Elective caesarean section (breech presentation)	Healthy female child of 3.3 kg	Potassium and Magnesium supplementation	Oligohydramnios
McCarthy et al. [14]	Postural hypotension, severe fatigue	2.6 mmol/L	Cesarean section (failure to progress in 1ststage of labour)	Healthy female child of 2.9 kg	Amiloride, Intravenous Potassium and Magnesium supplementation. Required 39 hospitalizations	Oligohydramnios
Raffi et al. [15]	Severe fatigue	3 mmol/L	Cesarean section	Healthy male child of 3 kg	Potassium and Magnesium supplementation	Fetal macrosomia (patient had gestational diabetes mellitus)
Morton et al. [16]	Lethargy, Muscle cramps, nausea, thirst, nocturia	2.6 mmol/L	Vaginal Delivery	Healthy female child of 3.6 kg	Potassium and Magnesium supplementation	None
Present Study	Absent fetal movements, loose stools, vomiting	1.90 mEq/L	Emergency caesarean section	Intra-uterine fetal demise	Oral Potassium and Magnesium supplementation	Intrauterine fetal demise

## Conclusions

Gitelman syndrome in pregnancy is a common but overlooked cause of hypokalemia in pregnancy which can have a grave effect on the fetomaternal outcome. Due to its varied presentations, it is often misdiagnosed and hence not appropriately managed. Gitelman's syndrome diagnosis must be made with a valid index that is very accurate, followed by meticulous monitoring and aggressive care, for a better prognosis of the mother and the foetus.
